# Long-term clinical outcomes and health-related quality of life in patients with isolated methylmalonic acidemia after liver transplantation: experience from the largest cohort study in China

**DOI:** 10.1007/s12519-023-00780-0

**Published:** 2024-01-08

**Authors:** Yi-Zhou Jiang, Guang-Peng Zhou, Lin Wei, Wei Qu, Zhi-Gui Zeng, Ying Liu, Yu-Le Tan, Jun Wang, Zhi-Jun Zhu, Li-Ying Sun

**Affiliations:** 1 Department of Critical Liver Diseases, Liver Research Center, Beijing Friendship Hospital, Capital Medical University, No. 101 Lu Yuan Dong Road, Tong-Zhou District, Beijing, 110112 China; 2 Liver Transplantation Center, National Clinical Research Center for Digestive Diseases, Beijing Friendship Hospital, Capital Medical University, Beijing, China; 3 Clinical Center for Pediatric Liver Transplantation, No. 101 Lu Yuan Dong Road, Tong-Zhou District, Capital Medical University, Beijing, 110112 China

**Keywords:** Health-related quality of life, Inborn error of metabolism, Isolated methylmalonic acidemia, Liver transplantation, Outcomes

## Abstract

**Background:**

Liver transplantation (LT) has been proposed as a viable treatment option for selected methylmalonic acidemia (MMA) patients. However, there are still controversies regarding the therapeutic value of LT for MMA. The systematic assessment of health-related quality of life (HRQoL)-targeted MMA children before and after LT is also undetermined. This study aimed to comprehensively assess the long-term impact of LT on MMA, including multiorgan sequelae and HRQoL in children and families.

**Methods:**

We retrospectively evaluated 15 isolated MMA patients undergoing LT at our institution between June 2013 and March 2022. Pre- and post-transplant data were compared, including metabolic profiles, neurologic consequences, growth parameters, and HRQoL. To further assess the characteristics of the HRQoL outcomes in MMA, we compared the results with those of children with biliary atresia (BA).

**Results:**

All patients had early onset MMA, and underwent LT at a mean age of 4.3 years. During 1.3–8.2 years of follow-up, the patient and graft survival rates were 100%. Metabolic stability was achieved in all patients with liberalized dietary protein intake. There was a significant overall improvement in height *Z* scores (P = 0.0047), and some preexisting neurological complications remained stable or even improved after LT. On the Pediatric Quality of Life Inventory (PedsQL™) generic core scales, the mean total, physical health, and psychosocial health scores improved significantly posttransplant (*P* < 0.05). In the family impact module, higher mean scores were noted for all subscales post-LT, especially family function and daily activities (*P* < 0.01). However, the total scores on the generic core scales and transplant module were significantly lower (Cohen’s *d* = 0.57–1.17) when compared with BA recipients. In particular, social and school functioning (Cohen’s *d* = 0.86–1.76), treatment anxiety, and communication (Cohen’s *d* = 0.99–1.81) were far behind, with a large effect size.

**Conclusions:**

This large single-center study of the mainland of China showed an overall favorable impact of LT on isolated MMA in terms of long-term survival, metabolic control, and HRQoL in children and families. The potential for persistent neurocognitive impairment and inherent metabolic fragility requires long-term special care.

Video Abstract (MP4 153780 KB)

**Supplementary Information:**

The online version contains supplementary material available at 10.1007/s12519-023-00780-0.

## Introduction

Methylmalonic acidemia (MMA), an inborn error of metabolism, is one of the most common organic acidurias. The subtype isolated MMA is caused by the deficiency of methyl malonyl-CoA mutase enzyme (MMUT, OMIM #251000) and appears more severe [1, 2]. The onset age of affected individuals is usually within the first year after birth and even as early as the neonatal period. The clinical characteristics include episodic metabolic decompensation or stroke with acidosis, neurological complications, developmental delay, and lethargy, eventually leading to coma, with relatively high mortality [3]. Due to metabolic fragility and multiple-system involvement, isolated MMA patients always require frequent hospitalizations, which gravely compromise the health-related quality of life (HRQoL) of children and their families [4–7].

Conventional medical and dietary treatments can only bring limited effects for isolated MMA [3, 8]. Since the enzyme activity that helps convert circulating metabolites can be restored by replacing the diseased liver with a metabolically normal liver, liver transplantation (LT) appears to be a viable alternative therapeutic option for selected MMA patients [9]. Since the first attempt in 1997, LT has been performed as a kind of enzyme replacement therapy for an increasing number of patients to prolong survival and reduce the morbidity of complications [7, 10–12]. However, LT is not a complete cure due to systemic metabolic defects. In some cases, acute metabolic decompensations and long-term complications still occur after LT [13, 14]. Given the rarity of MMA, it is difficult to reach a consensus on the clinical outcomes following LT, thereby leading to the ongoing controversy about the therapeutic value of LT. Therefore, more detailed long-term follow-up studies that can provide evidence for outcome parameters are necessary.

With advances in surgical techniques and perioperative and long-term postoperative management, survival is no longer the only goal in the long run, especially for pediatric recipients with inherited metabolic liver diseases. More in-depth health status assessments, including LT- or disease-related complications, patient-reported outcomes, and social function, are needed to fully evaluate the effectiveness of LT and guide physicians in timely therapeutic interventions. According to the newly published guidelines for the diagnosis and management of MMA, HRQoL, as an outcome parameter second only to survival, has been highly rated by health professionals, experts, and patient representatives [15]. However, there was no systematic assessment of HRQoL-targeted MMA children before and after LT. Hence, a comprehensive evaluation of the long-term effects of LT on MMA sufferers is urgently needed.

Here, this study systematically evaluates the long-term impact of LT on isolated MMA patients, including metabolic control, disease-specific multiorgan sequelae, and HRQoL (using Pediatric Quality of Life Inventory, PedsQL™ [16]), both in children and families, aiming to provide a comprehensive understanding of clinical outcomes in isolated MMA patients after LT. Furthermore, we compared the HRQoL outcomes in MMA with biliary atresia (BA) recipients to better illustrate the characteristics of this rare metabolic disease.

## Methods

### Study population

Patients diagnosed with isolated MMA who underwent LT at our hospital between June 2013 and March 2022 were identified from the patient database and enrolled in this study (including seven cases described previously [17]). All patients had at least one year of follow-up. Individuals without a confirmed molecular or biochemical diagnosis were excluded. The study was approved by the Ethics Committee Review Board of Beijing Friendship Hospital, Capital Medical University, and conducted according to the ethical guidelines of the Declaration of Helsinki and the Declaration of Istanbul. Informed consent to participate in the study was obtained from the legal guardian of the participants.

### Data collection

We retrospectively collected demographic data, including sex, onset age, age of diagnosis, genetic analysis, and LT-related details (age at LT, donor type, graft type, operation time, length of hospital stay, and post-LT complications). Clinical data, including (1) clinical manifestation-episodes of metabolic stroke/decompensation/seizure/epilepsy, intermittent vomiting, vision, and hearing impairment, brain MRI imaging abnormalities, and cognitive function; (2) medical treatment; (3) metabolic profiles—urine organic acids, plasma amino acid measurements, and blood acylcarnitine profile; (4) laboratory measures- liver function, renal function, and hematological parameters; (5) body growth parameters—weight and height; and (6) dietary protein intake, were collected pre- and post-LT until the most recent follow-up visit.

Vision and hearing impairment can be diagnosed after a formal ophthalmologic assessment or hearing test. The evidence of cognitive impairment involves developmental delay, intellectual disability, and/or behavioral abnormalities evaluated by healthcare professionals. Height- and weight-for-age *Z* scores were calculated based on the overall physical growth status of children in China [18]. The standard value is − 2 to 2 SD. A height *Z* score less than − 2 SD represents growth retardation, and a weight *Z* score less than − 2 SD means malnutrition.

### Pediatric quality of life inventory

A pediatric quality-of-life survey was conducted among all MMA patients. The questionnaires comprised the PedsQL™ 4.0 generic core scales, PedsQL™ 3.0 transplant module, and PedsQL™ 2.0 family impact module to measure HRQoL in children, parents, and families. They focus on the general aspects of physical, emotional, social, and school functioning; family functioning and transplant-specific functioning involve various dimensions. The parent proxy-report format was completed by parents during routine clinic visits according to the status before and after LT after informed consent.

The PedsQL™ generic core scales, containing 23 items, comprises four subscales: physical (8 items), emotional (5 items), social (5 items), and school functioning (5 items) [19]. The psychosocial health summary score is calculated as the sum of the last three subscales divided by the number of items (15).

The PedsQL™ family impact module, containing 37 items, comprises nine subscales: physical (6 items), emotional (5 items), social (4 items), and cognitive functioning (5 items) of the family; communication (3 items), worry (5 items), family daily activities (3 items), family relationships (5 items) and family financial burden (1 item) [20]. It is used to assess the impact of children's health conditions on parent and family functioning. The parent HRQoL summary score and family function summary score are computed accordingly.

The PedsQL™ transplant module*,* containing 46 items, comprises eight subscales: (1) medicines I (9 items, focus on the difficulty in taking medicines); (2) medicines II (8 items, focus on the difficulty in dealing with side effects of medication); (3) transplant and others (8 items, social and daily barriers LT brings about); (4) pain and hurt (3 items, body discomfort); (5) worry (7 items associated with health conditions after LT; (6) treatment anxiety (4 items, concerns of clinical visit/invasive medical practices); (7) perceived physical appearance (3 items, how I look) and (8)communication (4 items, with health care professionals) [16] were used to assess the impact of LT on children from different aspects.

All the instruments use a 5-point Likert scale (0 = never a problem, 1 = almost never a problem, 2 = sometimes a problem, 3 = often a problem, and 4 = almost always a problem) and are converted linearly into a 0–100 scale (0 = 100, 1 = 75, 2 = 50, 3 = 25, and 4 = 0) and averaged for each domain as the final score. Higher scores indicate better HRQoL. As an existing translation, the Chinese version of the scales was obtained on the PedsQL Web site (http://www.pedsql.org/translations.html). It has passed the linguistic validation process, and its availability and validity have been checked [21].

### Statistical analysis

Height and weight *Z* scores, mean levels of MMA and propinoylcarnitine (C3) (values of each subject were averaged for pre-LT and post-LT when multiple times of data exist), total and subscale scores of PedsQL™ generic core scales and family impact module pre- and post-LT were collected and compared by paired sample Student’s t test. Participants with no available pre- and/or post-LT data were excluded from the analysis.

To assess the differences in generic HRQoL and transplant-specific HRQoL between MMA and BA children after LT, we compared the data with two other papers from China [*n* = 51, 96% BA children, 1.02–7.03 years after living donor liver transplant (LDLT)] [22] and Japan (*n* = 74, 100% BA children, 0.4–17.5 years after LDLT) [23] using independent samples t tests. Effect sizes were calculated to measure the magnitude of the differences and are interpreted as small (0.20–0.49), moderate (0.50–0.79), and large (> 0.80) differences. All statistical analyzes were performed using Prism 9.0 software (GraphPad Software, San Diego, CA, USA) and R version 3.6.3. A *P* < 0.05 was considered statistically significant.

## Results

### Baseline characteristics of the patients

During June 2013 and March 2022, 15 patients diagnosed with isolated MMA underwent LT in our hospital (Table 1). All patients had early onset MMA, and the onset age ranged from one day to eight months after birth. The typical initial clinical manifestations included vomiting, poor feeding, lethargy, coma, and hypotonia. The diagnoses were confirmed by molecular genetic testing in all patients, and pathogenic variants of c.729_730insTT and c.914T>C in *MMUT* were the most frequent. Despite good compliance with a strict protein-restriction diet and levocarnitine supplementation, all patients required frequent hospitalizations due to metabolic acidosis or other disease-related complications, which fell into the indications for LT. One case (P11) asked for a preemptive LT to prevent disease progression when children were at an early age. Four patients suffered kidney dysfunction before surgery. All of the patients had developmental delays and intellectual disabilities.

**Table 1:** Baseline characteristics of 15 isolated MMA patients

No	Sex	Onset age	Disease type	Initial clinical manifestations	Gene	Variant 1	Variant 2	Indication for LT
1	F	5 d	Early-onset	Vomiting, poor feeding, anorexia, coma	*MMUT*	c.914T>C	c.1677-1G>A	Frequent MDs
2	F	8 d	Early-onset	Poor feeding, lethargy, recurrent vomiting	*MMUT*	c.914T>C	c.1880A>G	Frequent MDs
3	M	6 mon	Early-onset	Poor feeding, vomiting, seizures, and coma	*MMUT*	c.861G>C	c.1138G>A	Frequent MDs
4	M	8 mon	Early-onset	High fever with nausea and vomiting	*MMUT*	c.494A>G	c.729_730insTT	Frequent MDs
5	M	6 mon	Early-onset	Recurrent vomiting, poor feeding, hypotonia, lethargy, coma	*MMUT*	c.425C>T	c.1777G>T	Frequent MDs
6	M	3 d	Early-onset	Recurrent vomiting, lethargy	*MMUT*	c.398_399delGA	c.729_730insTT	Frequent MDs
7	F	3 d	Early-onset	Hypotonia, lethargy, hyperammonemia, diarrhea	*MMUT*	c.729_730insTT	c.2179C>T	Frequent MDs
8	M	1 d	Early-onset	Vomiting, dyspnea, acidosis	*MMUT*	c.91C>T	c.693C>A	Frequent MDs
9	M	7 d	Early-onset	Poor feeding, intermittent vomiting, lethargy, anemia	*MMUT*	c.2131G>T	c.1741C>T	Frequent MDs
10	M	18 d	Early-onset	Dyspnea, acidosis, diarrhea	*MMUT*	c.729_730insTT	c.1106G>A	Frequent MDs
11	M	3 d	Early-onset	Poor feeding, hypotonia, seizures, anemia	*MMUT*	c.1741C>T	c.419T>C	Preemptive treatment
12	M	7 mon	Early-onset	Vomiting, acidosis	*MMUT*	c.1718T>C	c.754-1G>C	Frequent MDs
13	M	3 d	Early-onset	Vomiting, lethargy, acidosis, coma	*MMUT*	c.1159A>C	c729_730insTT	Frequent MDs
14	F	40 d	Early-onset	Poor feeding, vomiting, lethargy, coma	*MMUT*	c.554C>T	c.729_730insTT	Frequent MDs
15	F	3 d	Early-onset	Poor feeding, vomiting, lethargy, acidosis, seizures, developmental delay	*MMUT*	c.1677-1G>A	c.914T>C	Frequent MDs

*MMA* methylmalonic acidemia or methylmalonic acid, *F* female, *M* male, *MDs* metabolic decompensations, *MRI* magnetic resonance imaging, *NA* not available, *Cr* creatinine

### Transplant procedures

Patients underwent LT at a mean age of 4.3 years [standard deviation (SD) 3.0]. Nine of 15 (60%) of the liver grafts were from living donors, while others were from deceased donors. The perioperative periods were uneventful except in one patient (P8). He underwent a partial enterectomy for a perforated colon secondary to bacterial infection and was successfully discharged on day 72 after LT. According to the standard protocols of our center [24], the immunosuppressive regimen was based on tacrolimus and low-dose corticosteroids, which were gradually tapered and withdrawn if clinical status permitted.

### Clinical outcomes

#### Liver trasplantation-related outcomes

At a median follow-up of 5.0 years (range 1.3–8.2), patient and graft survival rates were both 100%. Post-LT complications are summarized in Table 2. Three patients experienced mild pneumonia after LT. Cytomegalovirus (CMV) and Epstein‒Barr virus (EBV) infections were the most common infections, occurring in 53.3% (8/15) and 60% (9/15) of patients, respectively. All of them had asymptomatic viremia, with only the presence of virus in the blood. After reducing the immunosuppressive load and treating it with oral anti-viral medication, the viral load was reduced, thus preventing the direct and indirect effects of EBV/CMV. No one developed potentially life-threatening EBV-related posttransplant lymphoproliferative disease. Although three children had episodes of acute cellular rejection during follow-up, no histologic abnormality was observed in the latest liver biopsy. Twelve patients were on tacrolimus maintenance monotherapy for immunosuppression, while the remaining three recipients were readministered methylprednisolone because of mild acute rejection. Biliary (*n* = 2) and vascular complications (*n* = 1) were managed accordingly without severe consequences.

**Table 2:** LT-related information and pre/posttransplant clinical characteristics of MMA patients

No.	Age at LT (y)	Brain MRI imaging abnormalities	Type of LT	Follow-up (years)	Post-LT complications	Serum Cr (mg/dL)			Metabolic decompensation			Mean MMA (mmol/mol Cr)			Mean C3 (μmol/L)	
						Pre	Post		Pre	Post		Pre	Post		Pre	Post
1	8.1	Bilateral basal ganglia damages	Deceased	8.2	CMV + EBV +	0.18	0.50		Y	N		1160.6	293.4		NA	NA
2	2.6	White matter changes	Living	5.7	Pneumonia, EBV +	0.49	0.54		Y	N		601.6	212.4		35.77	37.82
3	11.2	None	Deceased	5.7	None	1.26^*^	1.60		Y	N		1453.3	58.6		69.08	24.12
4	7.1	Bilateral basal ganglia damage, delayed myelination	Deceased	5.6	Pneumonia, CMV + EBV +	0.86*	1.06		Y	N		759.9	353.2		62.43	32.73
5	2.2	NA	Deceased	5.5	Pneumonia, CMV + EBV +	0.67	0.68		Y	N		2384.9	363.0		80.35	30.62
6	6.0	Bilateral basal ganglia damages	Living	5.3	Acute rejection, CMV + EBV +	0.82^a^	1.15		Y	N		1387.9	167.9		41.93	26.46
7	4.0	Gliosis of bilateral frontal and parietal subcortical white matter	Living	5.2	CMV + EBV +	0.36	0.64		Y	N		3196.3	190.1		59.45	37.46
8	1.5	Delayed myelination	Living	5.0	Bile leakage, perforation of the colon, CMV + EBV +	0.42	0.41		Y	N		493.4	66.1		48.77	20.89
9	2.9	White matter changes, delayed myelination	Living	4.9	CMV +	0.68	1.16		Y	N		2747.1	67.7		46.8	22.21
10	6.9	NA	Living	4.8	Acute rejection	0.62	0.79		Y	N		898.9	407.0		32.29	24.83
11	0.4	Bilateral basal ganglia damages	Deceased	4.7	Hepatic artery occlusion	0.21	0.48		Y	N		823.3	390.0		74.11	39.16
12	3.7	None	Living	3.5	EBV +	0.35	0.57		Y	N		244.1	NA		30.9	NA
13	1.8	Bilateral basal ganglia damages	Living	3.3	Acute rejection, anastomotic stenosis, CMV + EBV +	0.95^a^	0.79		Y	N		164.8	NA		70.13	NA
14	3.9	Left basal ganglia damages	Deceased	1.8	None	0.38	0.60		Y	N		124.0	107.6		22.19	16.6
15	1.9	None	Living	1.3	Perforation of colon	0.24	0.27		Y	N		825.0	259.3		40.11	14.7

^a^Had preexisting kidney dysfunction before liver transplantation

*LT* liver transplantation, *MMA* methylmalonic acidemia or methylmalonic acid, *C3* propinoylcarnitine, *NA* not available, *CMV* cytomegalovirus, *EBV* Epstein‒Barr virus, *Y* yes, *N* no, *Cr* creatinine

### Metabolism

**Fig.1:**
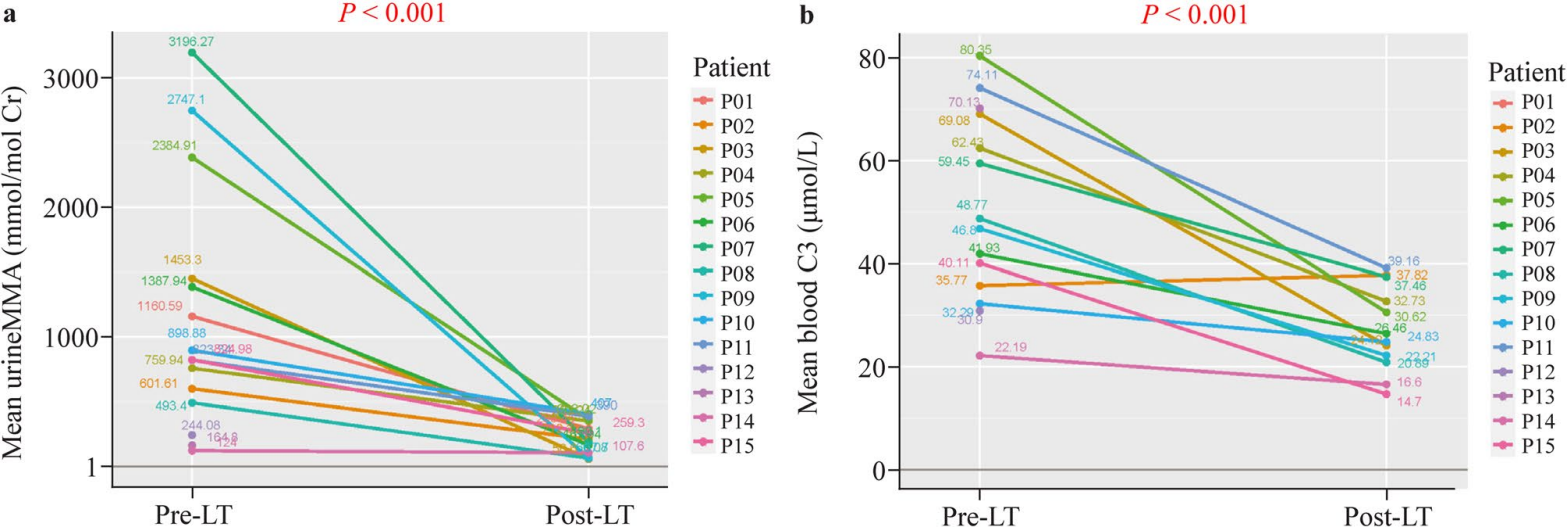
The mean levels of urine MMA and blood C3 before and after liver transplantation. **a** Mean urine MMA changes for the individual patient. Statistical analysis showed a significant reduction after surgery (pre-LT vs. post-LT, *P* < 0.001); **b** mean blood C3 changes for the individual patient. Statistical analysis showed a significant reduction after surgery (pre-LT vs. post-LT, *P* < 0.001). The normal range for C3: 1–4 µmol/L, MMA by GC–MS: 0.2–3.6 mmol/mol creatinine. *C3* propionylcarnitine, *LT* liver transplantation, *MMA* methylmalonic acidemia or methylmalonic acid, *GC–MS* gaschromatography-mass spectrometry

No recipients experienced episodes of metabolic stroke or decompensation requiring hospitalization throughout the follow-up period. The mean urine MMA level decreased from 1151.0 ± 941.7 mmol/mol creatinine before LT to 225.9 ± 128.4 mmol/mol creatinine, with statistical significance (*P* < 0.001, normal range: 0.2–3.6); the mean blood C3 also significantly decreased from 51.0 ± 18.3 to 27.3 ± 8.3 μmol/L (*P* < 0.001, normal range: 1.0–4.0) after surgery. Details for individual patients are shown in Table 2 and Fig. 1. The most recent clinical visit showed that dietary protein relaxation/liberalization was attained in all patients. Levocarnitine supplementation was also continued to ensure better metabolic stability.

### Neurological complications

Before LT, all patients presented with intermittent vomiting, and seizures occurred in four of them. Of the 13 children with available pre-LT data, 10 (76.9%) had abnormal brain magnetic resonance imaging (MRI), and basal ganglia lesions were the most common. For the other three patients, no lesion was found even after LT. Brain MRI abnormalities disappeared in one case (P11), who received an early LT in his fourth month. After LT, there was no occurrence of vomiting or seizures in any of the 15 patients. For other nervous system complications involving vision and hearing impairment (found in P6, P10, and P11), improvement was not obtained in any three of them. Different degrees of intellectual disability still existed postoperatively in all children but lacked detailed testing scores.

### Hematological complications

Thrombocytopenia and anemia were corrected in 2/2 (100%) and 9/14 (64.3%) patients, respectively. Another five recipients had persistent anemia after LT.

### Liver and kidney dysfunction

**Fig. 2:**
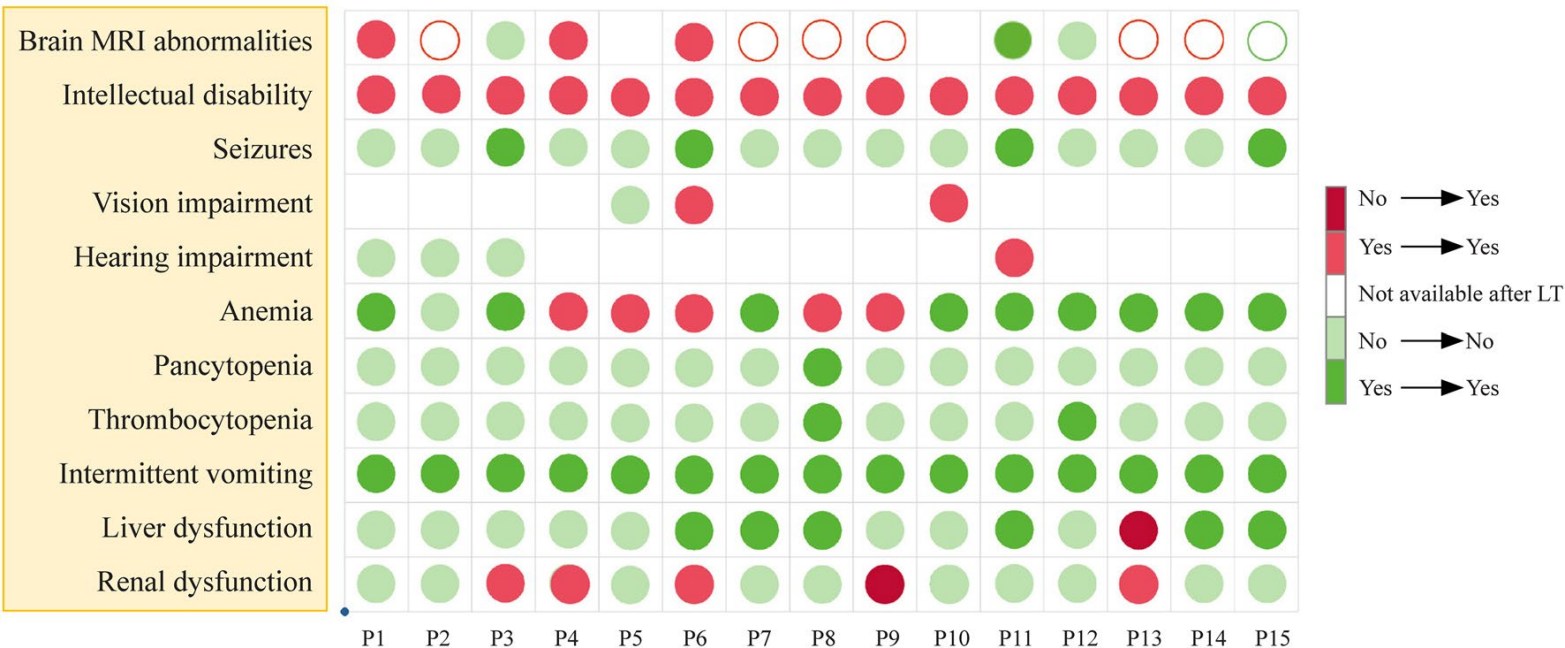
Clinical signs observed before and after liver transplantation in MMA patients. Filled dark red circles indicate that the sign is new-onset after LT; filled light red circles indicate that the sign consistently exists; filled dark green circles indicate that the sign is eliminated after LT; filled light green circles mean that the sign does not exist before and after LT; hollow light red/green circles mean that the sign does/does not exist before LT, while the data are unavailable after LT. *LT* liver transplantation, *MMA* methylmalonic acidemia

Among four patients suffering kidney dysfunction before LT, three worsened during the follow-up period, while the other improved (P13). Kidney impairment also newly developed in one child after LT (P9). Liver damage was remedied in all six children. The changes in clinical signs and symptoms before and after LT are shown in Fig. 2.

### Growth

**Fig. 3:**
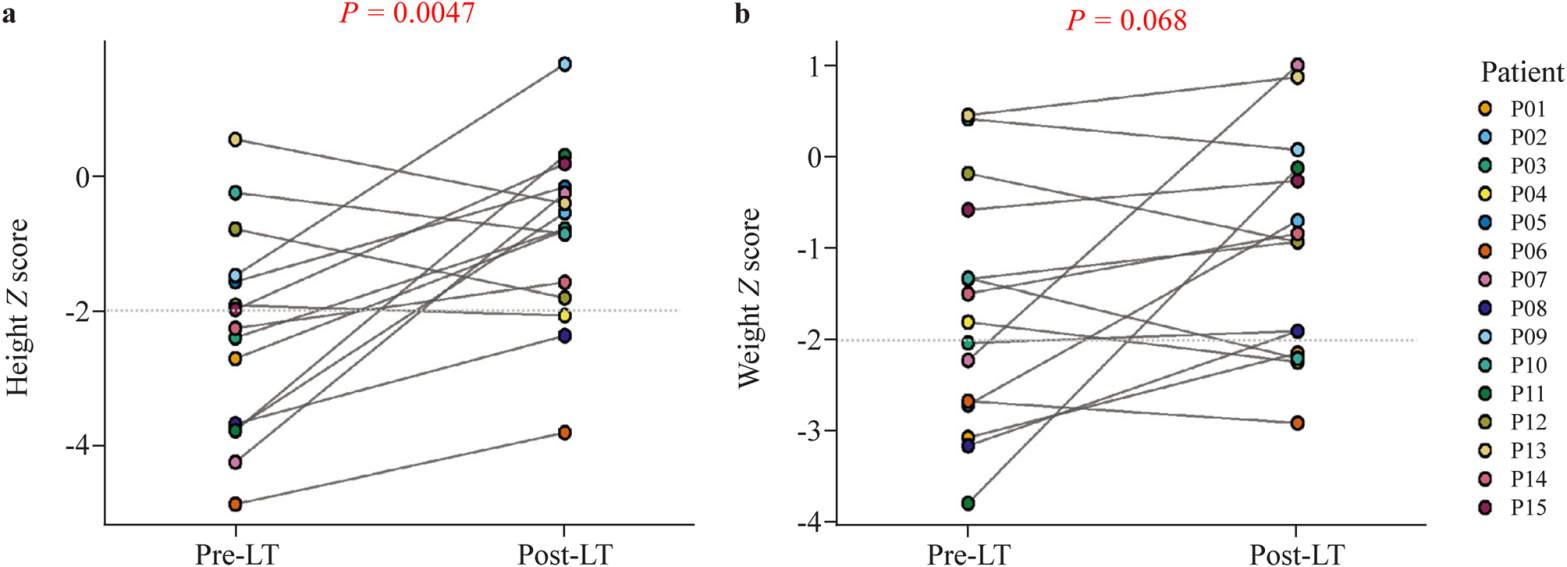
Height- and weight-for-age *Z* scores pre- and post-LT in MMA patients. **a** Statistical analysis showed a significant increase in height Z score after surgery (pre-LT vs. post-LT, *P* = 0.0047); **b** statistical analysis did not show a significant increase in weight Z score after surgery (pre-LT vs. post-LT, *P* = 0.068). The gray dashed lines indicate the reference value of − 2 SD. *LT* liver transplantation, *MMA* methylmalonic acidemia, *SD* standard deviation

A significant increase in height *Z* score was achieved after LT (− 2.3 ± 1.5 SD pre-LT vs. − 0.9 ± 1.3 SD post-LT, *P* = 0.0047). Notably, among eight patients who showed growth retardation (*Z* score <  − 2 SD) before LT, 6 (75%) were restored to the normal range (− 2 SD to 2 SD). In addition, an increasing trend was observed in weight *Z* scores, although without significant differences (− 1.7 ± 1.3 SD pre-LT vs. − 1.0 ± 1.2 SD post-LT, *P* = 0.068). Among 8 children who suffered from growth retardation or malnutrition problems, five (62.5%) showed significant improvement after LT. However, there were still 5 patients who failed to be comparable with age-matched peers, including two who were newly onset (Fig. 3).

### Health-related quality of life

#### Impact of liver transplantation on HRQoL in methylmalonic acidemia children and families

**Fig. 4:**
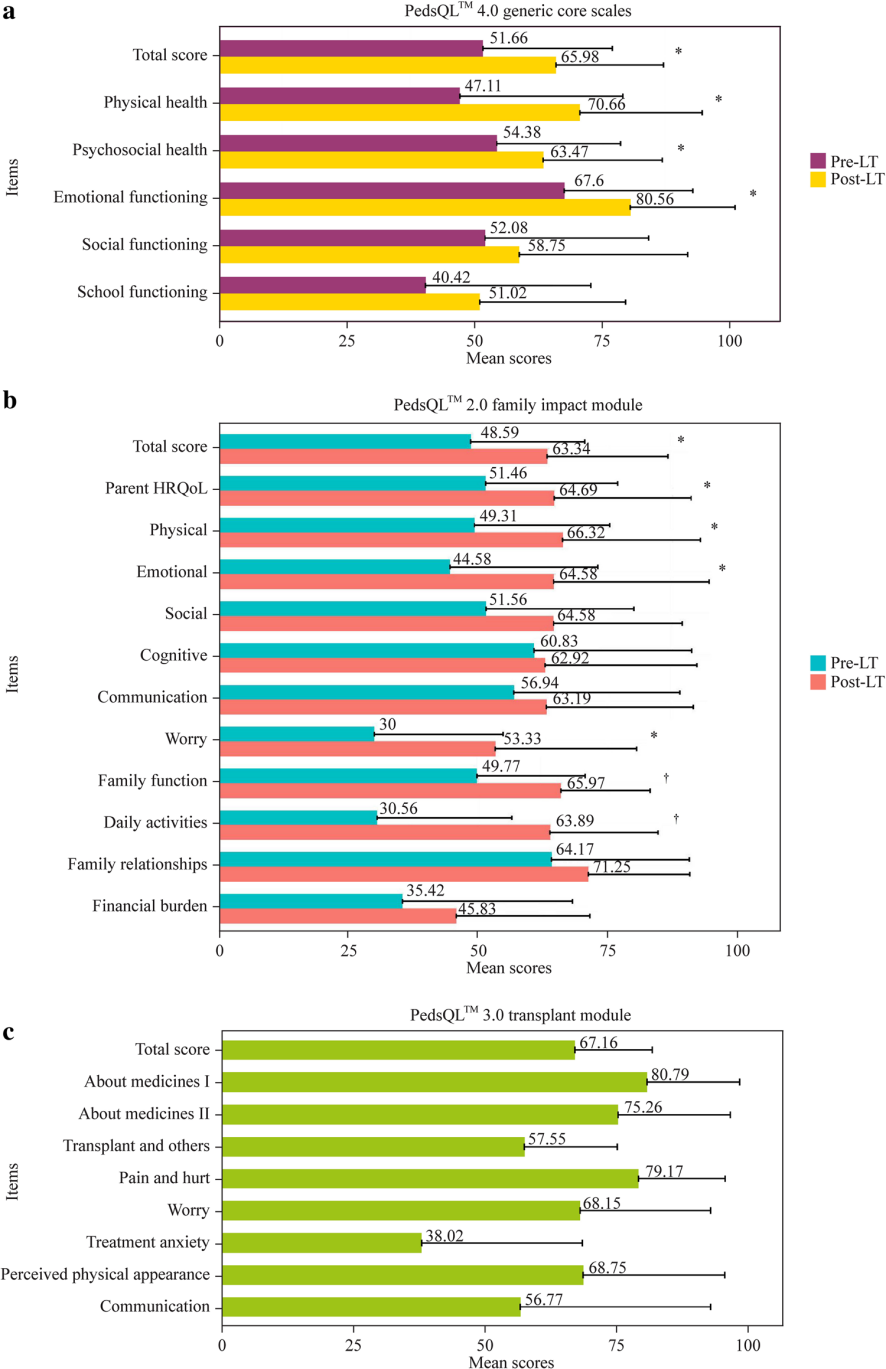
Parent proxy report for the Pediatric Quality of Life Inventory™ (PedsQL™) pre- and post-LT in MMA patients. Comparison of scores for **a** PedsQL™ 4.0 Generic Core Scales (*n* = 12); **b** PedsQL™ 2.0 Family Impact Module pre- and post-LT (*n* = 12); and **c** PedsQL™ 3.0 Transplant Module (*n* = 13). *P < 0.05, ^†^*P* < 0.01. *MMA* methylmalonic acidemia, *LT* liver transplantation, *HRQoL* health-related quality of life

To assess the impact of LT on HRQoL in the MMA population, mean total and subscale scores of the PedsQL™ generic core scales, family impact module, and transplant module (parent proxy report) pre- and post-LT were compared (Fig. 4). Twelve parents completed the first two scales, and 13 completed the transplantation-specific questionnaire, with no missing items in any response. On the PedsQL™ generic core scales, the mean scores improved in all domains, of which total (65.98 vs. 51.66, *P* = 0.03), physical health (70.66 vs. 47.11, *P* = 0.04) and psychosocial health sum scores (63.47 vs. 54.38, *P* = 0.03) were significantly higher after LT. Regarding subscales of psychosocial health, the score of emotional functioning reached 80.56 (vs. 67.60, *P* = 0.047). School and social functioning gained the two lowest scores, 51.02 ± 28.57 and 58.75 ± 33.04, respectively. On the family impact module, higher mean scores in each subscale after LT were noted, especially the total score (63.34 vs. 48.59, *P* = 0.02), parent sum HRQoL (64.69 vs. 51.46, *P* = 0.02), worry (53.33 vs. 30.00, *P* = 0.01), and family function sum score (65.97 vs. 49.77, *P* = 0.006), all of which improved with significant differences. Specifically, notable progress has been made in the family's physical and emotional function and daily activities. Among all dimensions, the scores of financial burden and worry were the lowest. For the PedsQL™ transplant module, the mean total score of these subjects was 67.16 ± 14.63. Domains that did not reach the value included transplant and others (mean = 57.55), treatment anxiety (mean = 38.02), and communication (mean = 56.77) (see details in Supplementary Table 1).

#### Comparison of HRQoL between children transplanted for methylmalonic acidemia and biliary atresia

The total score of PedsQL™ generic core scales in subjects with MMA after LT (65.98 ± 21.11) was significantly lower than BA children reported in Japan (80.2 ± 16.0, Cohen’s d = 0.85) and China (82.21 ± 13.71, Cohen’s *d* = 1.06), both with a large effect size. Physical health scored lower with a small to moderate effect size (Cohen’s *d* = 0.30–0.76). In the domain of psychosocial health, emotional functioning was comparable with the other two studies, while social (Cohen’s *d* = 1.07–1.17) and school functioning (Cohen’s *d* = 0.86–1.76) were still far behind. The overall score of the transplant module was rated lower in children with MMA than in those with BA, and the differences generated moderate effect sizes (Cohen’s *d* = 0.57–0.68). For the subscales, MMA patients had significantly lower scores in treatment anxiety and communication (Cohen’s *d* > 0.8). Compared with BA patients, the score in transplant and others was also considerably lower, but other domains displayed similar results (Table 3).

**Table 3:** Post-LT Pediatric Quality of Life Inventory™ (PedsQL™, Parent-Proxy) scores compared between children undergoing transplant for MMA and BA

Scale	Number of items	Present (MMA)		Japanese children (BA)			[23]	Chinese children (BA)			[22]
		Mean	SD	Mean	SD	Mean difference	Effect size	Mean	SD	Mean difference	Effect size
Number of participants		12		74				51			
*Generic Core Scales*											
Total score	23	65.98	21.11	80.2	16.0	− 14.22^†^	− 0.85	82.21	13.71	− 16.23^†^	− 1.06
Physical health	8	70.66	23.95	86.0	19.6	− 15.34*	− 0.76	77.45	22.60	− 6.79	− 0.30
Psychosocial health	15	63.47	23.37	−	−	−	−	83.79	13.08	− 20.32^‡^	− 1.32
Emotional functioning	5	80.56	20.50	79.5	17.7	1.06	0.06	80.78	19.35	− 0.22	− 0.01
Social functioning	5	58.75	33.04	81.2	18.6	− 22.45^‡^	− 1.07	83.24	17.26	− 24.49^‡^	− 1.17
School functioning	5	51.02	28.57	70.4	21.4	− 19.38^†^	− 0.86	87.35	18.48	− 36.33^§^	− 1.76
*Transplant module*											
Total score	46	67.16	14.63	75.8	12.3	− 8.64*	− 0.68	75.90	15.44	− 8.74	− 0.57
About medicines I	9	80.79	17.63	87.2	13.2	− 6.41	− 0.46	82.80	16.62	− 2.01	− 0.12
About medicines II	8	75.26	21.42	87.9	14.2	− 12.64^†^	− 0.82	75.40	19.76	− 0.14	− 0.01
Transplant and others	8	57.55	17.60	68.4	18.3	− 10.85	− 0.60	75.03	19.53	− 17.48^†^	− 0.91
Pain and hurt	3	79.17	16.48	88.0	17.1	− 8.83	− 0.52	80.23	18.56	− 1.06	− 0.06
Worry	7	68.15	24.77	55.7	21.1	12.45	1.14	68.28	25.22	− 0.13	− 0.01
Treatment anxiety	4	38.02	30.56	75.3	18.5	− 37.28^§^	− 1.81	66.42	28.20	− 28.40^†^	− 0.99
Perceived physical appearance	3	68.75	26.86	63.3	23.8	5.45	0.22	75.16	26.06	− 6.41	− 0.24
Communication	4	56.77	36.10	76.7	17.5	− 19.93^†^	− 0.94	83.82	17.77	− 27.05^‡^	− 1.20

Effect sizes are designated as small (0.20), medium (0.50), and large (0.80)

*LT* liver transplantation, *MMA* methylmalonic acidemia or methylmalonic acid, *BA* biliary atresia, *SD* standard deviation

*P < 0.05, ^†^*P* < 0.01, ^‡^*P* < 0.001, ^§^*P* < 0.0001

## Discussion

In this study, we retrospectively investigated the composite metrics of 15 isolated MMA patients who underwent LT in our institution. We found that LT is a safe and efficient solution to prolong survival and improve metabolic stability. Furthermore, it can also significantly ameliorate the impaired overall quality of life for patients and their families. Physical health and emotional functioning of children and parents and family function improved considerably after LT. However, compared with pediatric recipients with non-metabolic diseases, the consistently low social and school functioning and high levels of treatment anxiety, possibly related to the disease property, still require much attention in the long run.

The Mut0 type of MMA is a rare inborn error of mitochondrial metabolism with potentially lethal consequences and devastating multisystem damage. Conventional treatment always fails to be effective for this type of MMA [3, 25], and LT/combined liver and kidney transplantation has been increasingly conducted as an enzyme replacement therapy. In our series, patient and graft survival rates were satisfactory over a follow-up period of 1.3–8.2 years after LT. This is consistent with our recently published meta-analysis, where the pooled estimated survival rate was nearly 100% [26]. Although our MMA patients developed some LT-related complications, particularly EBV infection, CMV infection, biliary complications, and rejection, patients with MMA are not at significantly increased risk of complications compared to pediatric recipients with other primary liver diseases [27, 28]. Consequently, the safety of LT for MMA is well guaranteed, even when using partial liver grafts from asymptomatic heterozygous carrier donors [29].

As previously reported, most recipients achieved metabolic stability after LT [26]. In our cohort, the eradication of decompensation episodes after LT was observed in all patients. Although the levels are still far beyond the normal range, the reduction rates of accumulated metabolites reached 80.40% in urinary MMA and 46.50% in blood C3, both with significant differences. Frequent metabolic decompensations may lead to brain lesions and subsequent neurological damage. Brain MRI imaging abnormalities in MMA are characterized by hyperintensity on T2 weighted imaging (T2WI) and fluid attenuated inversion recovery (FLAIR) sequences in the basal ganglia during acute decompensation, delayed myelination, and white matter damage during chronic periods [15, 30]. In our study, no worsening or new onset brain damage was observed in any patients, and the lesions even resolved in one patient who received a timely LT as early as four months of age. In addition, patients with the early-onset mut0 subtype and frequent metabolic decompensations usually have more neurological complications, including optic neuropathy and sensorineural hearing loss [31, 32]. Different degrees of intellectual disability/cognitive development delay are common even after LT. However, based on our findings, further deterioration is unlikely to occur, and newly diagnosed abnormalities are rarely seen after LT. This provides additional evidence that LT can alter the natural course of MMA. Prompt diagnosis and early LT before the occurrence of irreversible neurological damage are strongly recommended. However, preemptive treatment requires comprehensive and multidisciplinary evaluation and adequate communication with parents.

Adequate daily intake of essential and functional amino acids is necessary for normal body growth in children. Thus, it is crucial for MMA patients to limit natural proteins to reduce metabolic toxicity while ensuring the basic physiological requirements of the precursor amino acids (isoleucine, methionine, valine, and threonine). Concerning the dietary management of patients with organic acidemia, no consensus on the protein intake amount after LT has been reported. Persistent dietary restriction was seen in some cases, while protein liberalization has been more frequently observed in metabolically stable children [33–35]. In our study, relaxed protein intake was achieved in all patients without episodes of metabolic decompensation. Meanwhile, poor growth is another problem affecting daily life in MMA along with dietary restrictions. After LT, more than 60% of our patients got rid of growth retardation/malnutrition at the last clinical visit. However, failure to thrive seems to be associated with multiple factors aside from nutritional reasons, as five children still suffered persistent growth delay. Therefore, regular individualized nutritional management and development monitoring under the guidance of pediatrists/metabolic dietitians in the long term are appropriate for MMA patients after LT. Notably, stroke or seizures occasionally occur in some reported cases, possibly due to the failure of the implanted liver graft to correct de novo propionyl-CoA and accumulated MMA in the central nervous system [7, 13]. Therefore, levocarnitine administration is still necessary after LT [15].

The HRQoL of children with MMA mut0 is substantially impaired [5]. HRQoL has been ranked as the second most crucial outcome parameter by health professionals, experts, and patient representatives according to the latest guidelines [15]. Thus, a comprehensive assessment of long-term health status, including HRQoL, is necessary for MMA patients and their families. However, to date, there is still a lack of structured quantitative measures to capture the impact of LT on HRQoL concerning the MMA population. By using specific instruments PedsQL™ to compare each patient’s HRQoL before and after LT, we demonstrated an overall significant improvement in physical and psychosocial health both in children and parents. Family function, particularly daily activities, was ameliorated after LT. We speculate that the elimination of metabolic decompensations and the prevention of the progression of disease-related complications by LT could reduce the frequency of hospitalization and alleviate parental stress, which contributes to positive patient-reported outcomes. Moreover, the financial burden on families was surprisingly relieved after LT. This unexpected finding subjectively verifies the previous report that LT is more cost-effective than conventional management (direct and indirect costs in an expected lifetime) [4].

We found that children with MMA always experience the greatest difficulties with social and school functioning (with the lowest score on the scale) before LT, consistent with a previous study [5]. Poor performance does not change much after LT and is far behind peers with nonmetabolic disease. Meanwhile, we found that treatment anxiety and communication with clinicians had the most significant negative impacts on the transplant module and were specific to MMA. Most parents reported that their children have difficulty expressing themselves clearly. The deficiency in social interactions may be attributed to disease-exclusive neurological damage. In MMA, intellectual abilities and cognitive impairment require a multidisciplinary team involving physical and rehabilitation therapists and pediatric neurologists for regular evaluation [3]. Linguistic training and other special education may be necessary in the long run. Another surprising finding is that MMA children displayed extreme fears regarding clinical visits and invasive medical procedures compared to BA recipients. The clinical symptoms of acute metabolic decompensation can be triggered by some situations, including medication (e.g., high-dose glucocorticoids), invasive procedures/general anesthesia, and psychological stress [15]. Hence, we assume that fears are a kind of self-defense against disease-specific metabolic brittleness in MMA. More understanding and comfort should be given to these patients.

Several limitations associated with our study should be discussed. First, due to the rarity of MMA, this was a single-center study with a small sample size, and thus, further analysis of the factors influencing post-LT outcomes could not be undertaken. Comparisons between these subjects and patients who did not undergo LT were also unavailable. Second, considering the disease-related intellectual disability and young age of the patients, HRQoL questionnaires were completed by their parents, and the results may not reflect the actual situation of the patients. Third, the comparison group of BA children was from other studies. However, to reduce bias, we selected two Asian populations, including one from China. Despite these limitations, we believe that our research provides clinicians and patients with the most comprehensive clinical evidence regarding the long-term outcomes of LT in treating MMA because it represents one of the largest single-center cohort studies in the world. More importantly, we adopted a more comprehensive assessment approach to evaluate the efficacy of LT for MMA from a multidimensional perspective.

In conclusion, our study reveals an overall favorable impact of LT on MMA mut0 patients. The procedure not only ensures long-term survival, metabolic stability, and prevention of multiorgan sequelae deterioration but also significantly improves the HRQoL in both children and families. In particular, we found significant improvements in physical and emotional functioning, but school and social functioning still fall behind, incomparable with nonmetabolic LT recipients. Most importantly, LT is not a cure for MMA. The potential persistent neurocognitive impairment and inherent metabolic brittleness make MMA recipients require a multidisciplinary team and long-term special care after LT. Early transplant in infancy seems to have a better outcome. Meanwhile, upon preparation of this paper, there are four new genomic therapies for MMA in phase 1–2 clinical trials. It is foreseeable that our data will be most important for the proper use of genomic therapies at the horizon.

## Supplementary Information

Below is the link to the electronic supplementary material.

Supplementary file1 (DOCX 23 KB)

## Data Availability

Data sharing not applicable to this article as no datasets were generated or analyzed during the current study.
