# Supplementary material for: Long-term clinical outcomes and health-related quality of life in patients with isolated methylmalonic acidemia after liver transplantation: experience from the largest cohort study in China
Source: World J Pediatr. 2024 Jan 8;20(8):809–21. doi: 10.1007/s12519-023-00780-0 (PMC11402840; doi:10.1007/s12519-023-00780-0)
Supplement: Supplementary file 2 — Supplementary file1 (DOCX 23 KB) [file 12519_2023_780_MOESM1_ESM.docx]

**Long-term Clinical Outcomes and Health-related Quality of Life in Patients with Isolated Methylmalonic Acidemia after Liver Transplantation: Experience from the Largest Cohort Study in China**

**Supplementary materials**

**Table S1 Pediatric Quality of Life Inventory^TM^ (PedsQL^TM^) scores of MMA patients after LT**

|  | Pre-LT | | | Post-LT | | |  |
| --- | --- | --- | --- | --- | --- | --- | --- |
|  | n | Mean | SD | n | Mean | SD | *P* value |
| Generic Core Scales |  |  |  |  |  |  |  |
| Total score | 12 | 51.66 | 25.39 | 12 | 65.98 | 21.11 | 0.03 |
| Physical health | 12 | 47.11 | 31.96 | 12 | 70.66 | 23.95 | 0.04 |
| Psychosocial health | 12 | 54.38 | 24.26 | 12 | 63.47 | 23.37 | 0.03 |
| emotional functioning | 12 | 67.60 | 25.22 | 12 | 80.56 | 20.50 | 0.05 |
| social functioning | 12 | 52.08 | 32.08 | 12 | 58.75 | 33.04 | 0.27 |
| school functioning | 12 | 40.42 | 32.37 | 12 | 51.02 | 28.57 | 0.08 |
| Transplant module |  |  |  |  |  |  |  |
| Total score | - | - | - | 13 | 67.16 | 14.63 | - |
| About medicines I | - | - | - | 13 | 80.79 | 17.63 | - |
| About medicines II | - | - | - | 13 | 75.26 | 21.42 | - |
| Transplant and others | - | - | - | 13 | 57.55 | 17.60 | - |
| Pain and hurt | - | - | - | 13 | 79.17 | 16.48 | - |
| Worry | - | - | - | 13 | 68.15 | 24.77 | - |
| Treatment anxiety | - | - | - | 13 | 38.02 | 30.56 | - |
| Perceived physical appearance | - | - | - | 13 | 68.75 | 26.86 | - |
| Communication | - | - | - | 13 | 56.77 | 36.10 | - |
| Family Impact Module |  |  |  |  |  |  |  |
| Total score | 12 | 48.59 | 21.99 | 12 | 63.34 | 23.24 | 0.02 |
| Parent HRQoL | 12 | 51.46 | 25.44 | 12 | 64.69 | 26.38 | 0.02 |
| physical | 12 | 49.31 | 26.10 | 12 | 66.32 | 26.56 | 0.01 |
| emotional | 12 | 44.58 | 28.48 | 12 | 64.58 | 29.96 | 0.02 |
| social | 12 | 51.56 | 28.47 | 12 | 64.58 | 24.76 | 0.12 |
| cognitive | 12 | 60.83 | 30.36 | 12 | 62.92 | 29.27 | 0.65 |
| Communication | 12 | 56.94 | 31.95 | 12 | 63.19 | 28.31 | 0.38 |
| Worry | 12 | 30.00 | 24.86 | 12 | 53.33 | 27.25 | 0.01 |
| Family function | 12 | 49.77 | 20.90 | 12 | 65.97 | 17.17 | 0.01 |
| daily activities | 12 | 30.56 | 25.95 | 12 | 63.89 | 20.82 | 0.01 |
| family relationships | 12 | 64.17 | 26.53 | 12 | 71.25 | 19.55 | 0.12 |
| financial burden | 12 | 35.42 | 32.78 | 12 | 45.83 | 25.75 | 0.13 |
